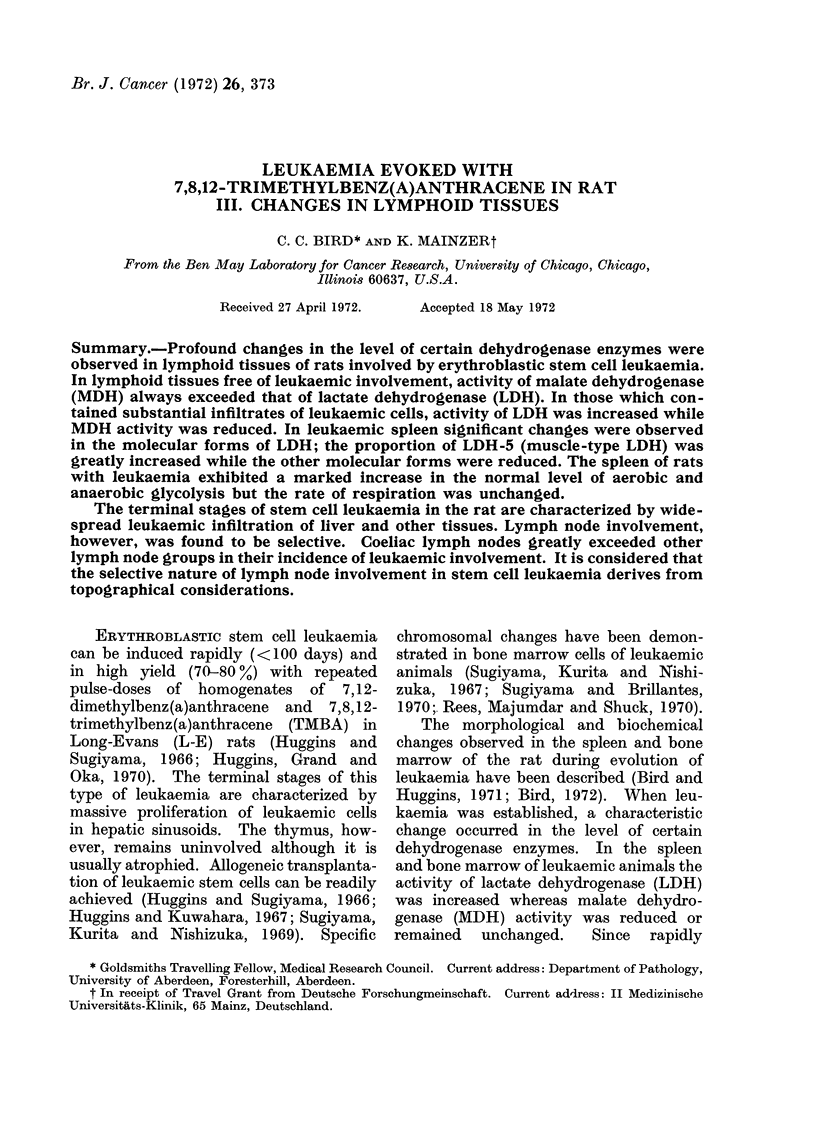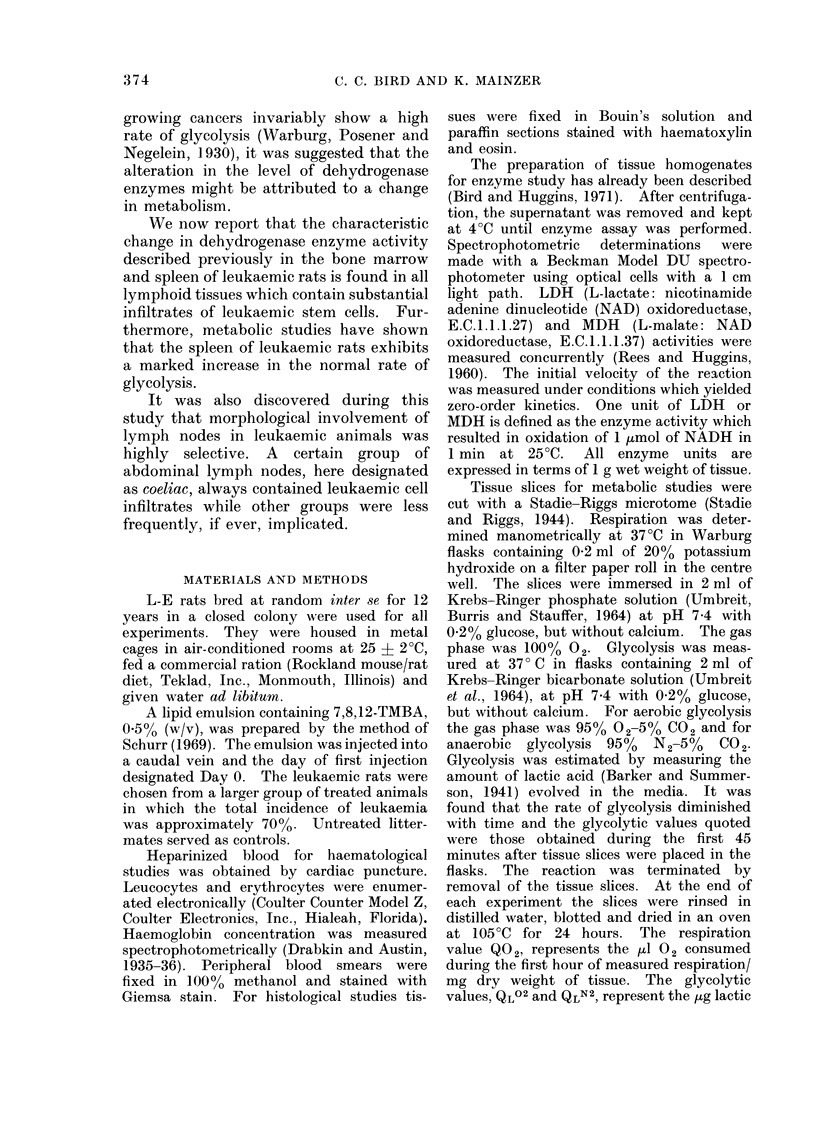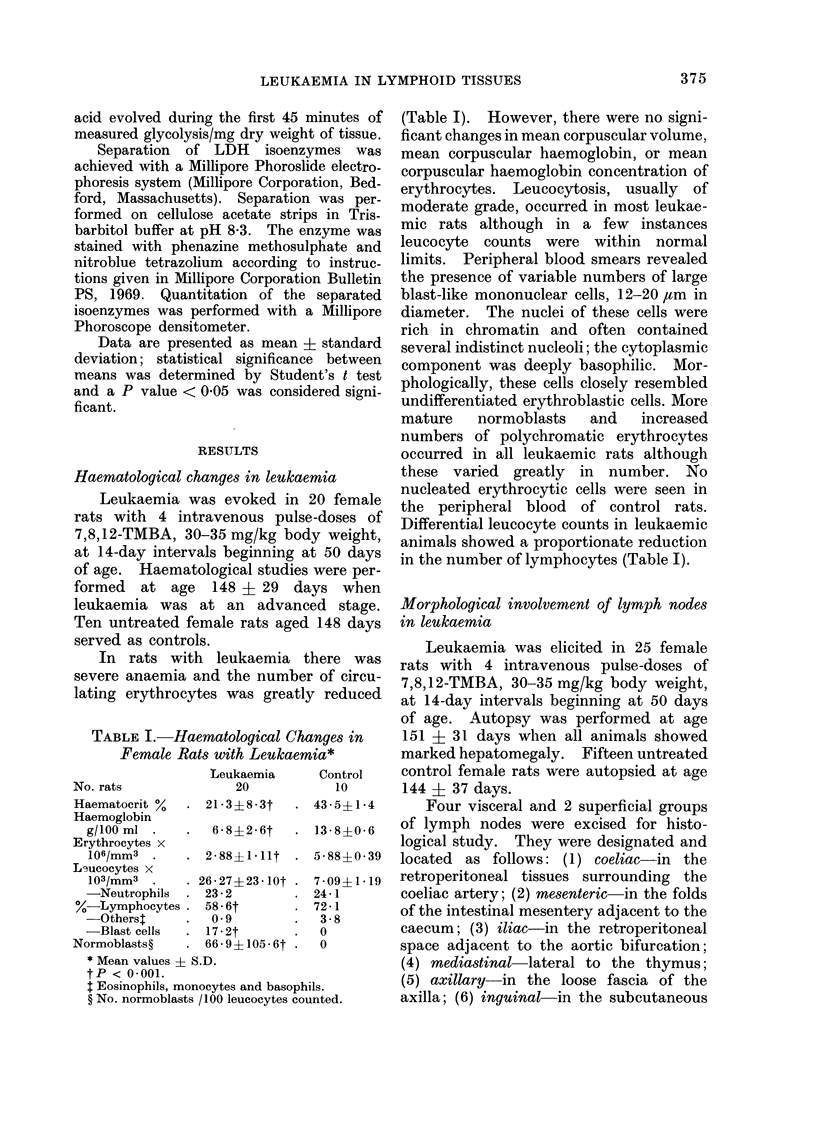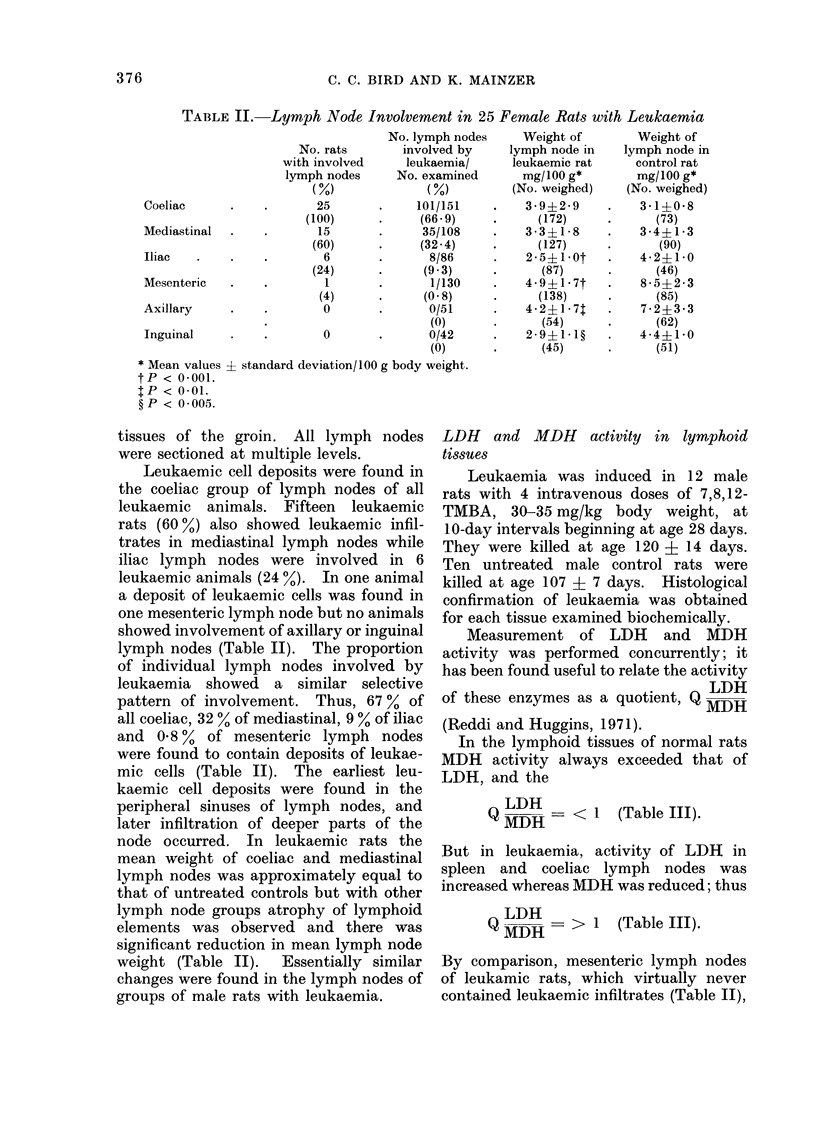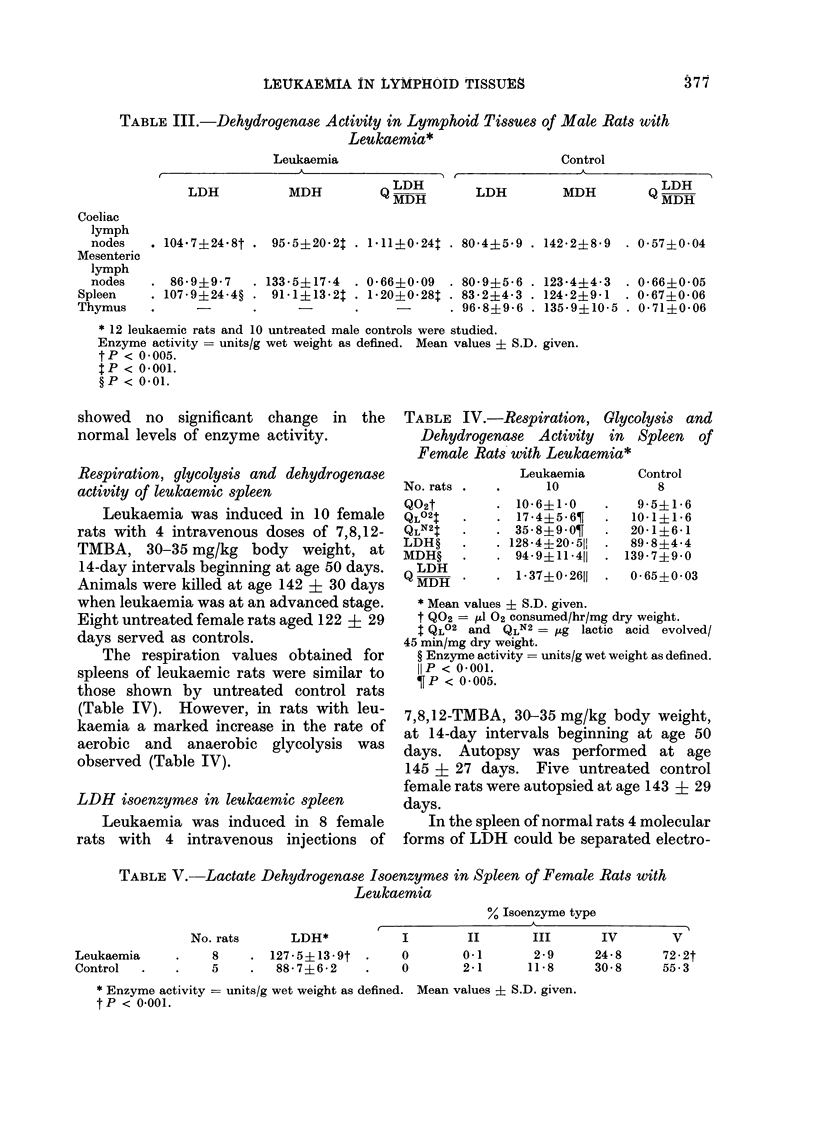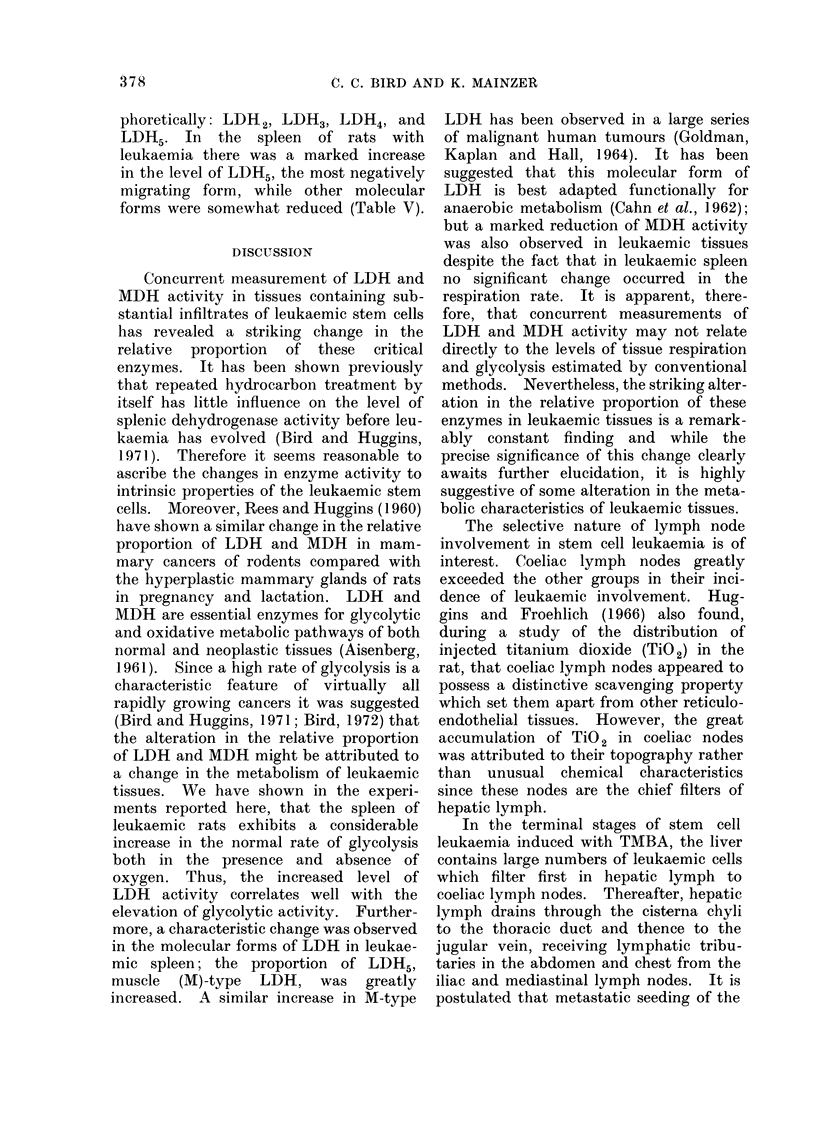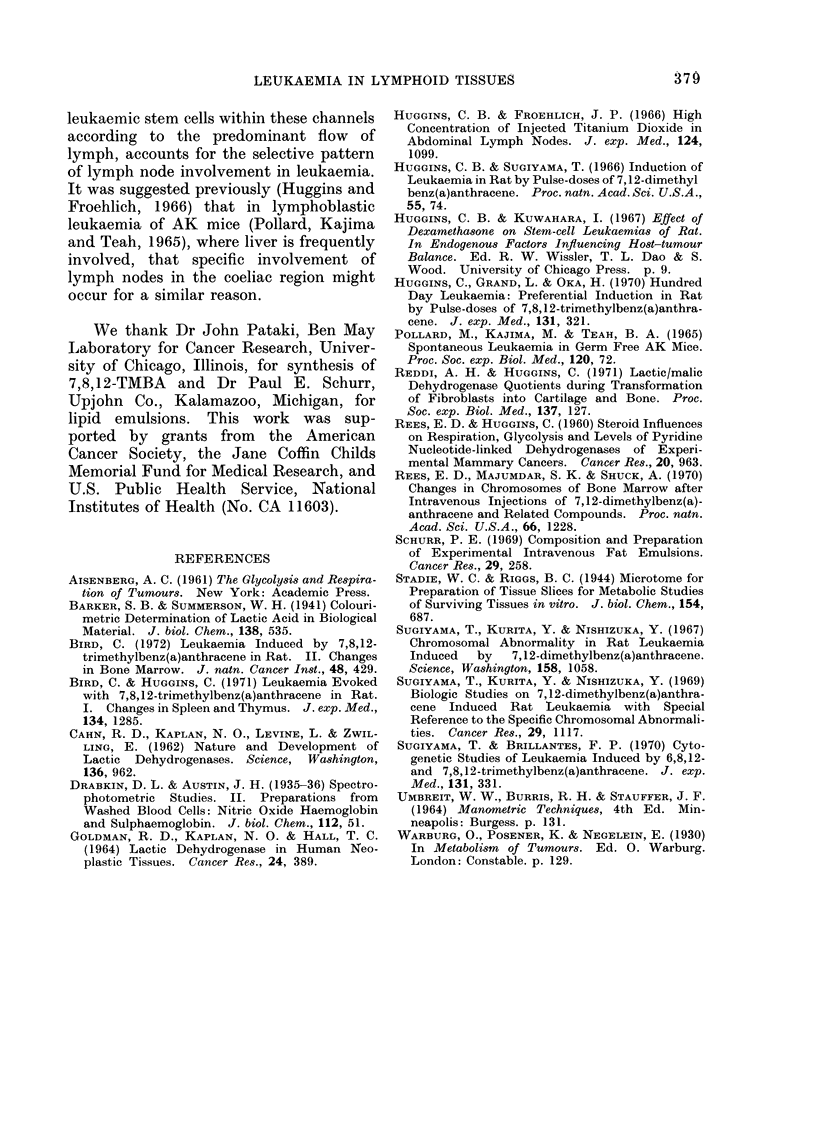# Leukaemia Evoked with 7,8,12-Trimethylbenz(a)Anthracene in Rat. III. Changes in Lymphoid Tissues

**DOI:** 10.1038/bjc.1972.49

**Published:** 1972-10

**Authors:** C. C. Bird, K. Mainzer

## Abstract

Profound changes in the level of certain dehydrogenase enzymes were observed in lymphoid tissues of rats involved by erythroblastic stem cell leukaemia. In lymphoid tissues free of leukaemic involvement, activity of malate dehydrogenase (MDH) always exceeded that of lactate dehydrogenase (LDH). In those which contained substantial infiltrates of leukaemic cells, activity of LDH was increased while MDH activity was reduced. In leukaemic spleen significant changes were observed in the molecular forms of LDH; the proportion of LDH-5 (muscle-type LDH) was greatly increased while the other molecular forms were reduced. The spleen of rats with leukaemia exhibited a marked increase in the normal level of aerobic and anaerobic glycolysis but the rate of respiration was unchanged.

The terminal stages of stem cell leukaemia in the rat are characterized by wide-spread leukaemic infiltration of liver and other tissues. Lymph node involvement, however, was found to be selective. Coeliac lymph nodes greatly exceeded other lymph node groups in their incidence of leukaemic involvement. It is considered that the selective nature of lymph node involvement in stem cell leukaemia derives from topographical considerations.


*Br. J. Cancer* (1972) **26**, 373

### LEUKAEMIA EVOKED WITH 7,8,12-TRIMETHYLBENZ(A)ANTHRACENE IN RAT III. CHANGES IN LYMPHOID TISSUES

C. C. BIRD* AND K. MAINZER†

*From the Ben May Laboratory for Cancer Research, University of Chicago, Chicago, Illinois* 60637, *U.S.A.*

Received 27 April 1972. Accepted 18 May 1972

**Summary.—Profound changes in the level of certain dehydrogenase enzymes were observed in lymphoid tissues of rats involved by erythroblastic stem cell leukaemia. In lymphoid tissues free of leukaemic involvement, activity of malate dehydrogenase (MDH) always exceeded that of lactate dehydrogenase (LDH). In those which contained substantial infiltrates of leukaemic cells, activity of LDH was increased while MDH activity was reduced. In leukaemic spleen significant changes were observed in the molecular forms of LDH; the proportion of LDH-5 (muscle-type LDH) was greatly increased while the other molecular forms were reduced. The spleen of rats with leukaemia exhibited a marked increase in the normal level of aerobic and anaerobic glycolysis but the rate of respiration was unchanged.**

**The terminal stages of stem cell leukaemia in the rat are characterized by widespread leukaemic infiltration of liver and other tissues. Lymph node involvement, however, was found to be selective. Coeliac lymph nodes greatly exceeded other lymph node groups in their incidence of leukaemic involvement. It is considered that the selective nature of lymph node involvement in stem cell leukaemia derives from topographical considerations.**

ERYTHROBLASTIC stem cell leukaemia can be induced rapidly (<100 days) and in high yield (70–80%) with repeated pulse-doses of homogenates of 7,12-dimethylbenz(a)anthracene and 7,8,12-trimethylbenz(a)anthracene (TMBA) in Long-Evans (L-E) rats (Huggins and Sugiyama, 1966; Huggins, Grand and Oka, 1970). The terminal stages of this type of leukaemia are characterized by massive proliferation of leukaemic cells in hepatic sinusoids. The thymus, however, remains uninvolved although it is usually atrophied. Allogeneic transplantation of leukaemic stem cells can be readily achieved (Huggins and Sugiyama, 1966; Huggins and Kuwahara, 1967; Sugiyama, Kurita and Nishizuka, 1969). Specific chromosomal changes have been demonstrated in bone marrow cells of leukaemic animals (Sugiyama, Kurita and Nishizuka, 1967; Sugiyama and Brillantes, 1970; Rees, Majumdar and Shuck, 1970).

The morphological and biochemical changes observed in the spleen and bone marrow of the rat during evolution of leukaemia have been described (Bird and Huggins, 1971; Bird, 1972). When leukaemia was established, a characteristic change occurred in the level of certain dehydrogenase enzymes. In the spleen and bone marrow of leukaemic animals the activity of lactate dehydrogenase (LDH) was increased whereas malate dehydrogenase (MDH) activity was reduced or remained unchanged. Since rapidly

* Goldsmiths Travelling Fellow, Medical Research Council. Current address: Department of Pathology, University of Aberdeen, Foresterhill, Aberdeen.

† In receipt of Travel Grant from Deutsche Forschungmeinschaft. Current address: II Medizinische Universitäts-Klinik, 65 Mainz, Deutschland.

growing cancers invariably show a high rate of glycolysis (Warburg, Posener and Negelein, 1930), it was suggested that the alteration in the level of dehydrogenase enzymes might be attributed to a change in metabolism.

We now report that the characteristic change in dehydrogenase enzyme activity described previously in the bone marrow and spleen of leukaemic rats is found in all lymphoid tissues which contain substantial infiltrates of leukaemic stem cells. Furthermore, metabolic studies have shown that the spleen of leukaemic rats exhibits a marked increase in the normal rate of glycolysis.

It was also discovered during this study that morphological involvement of lymph nodes in leukaemic animals was highly selective. A certain group of abdominal lymph nodes, here designated as *coeliac*, always contained leukaemic cell infiltrates while other groups were less frequently, if ever, implicated.

### MATERIALS AND METHODS

L-E rats bred at random *inter se* for 12 years in a closed colony were used for all experiments. They were housed in metal cages in air-conditioned rooms at 25 $\pm$ 2°C, fed a commercial ration (Rockland mouse/rat diet, Teklad, Inc., Monmouth, Illinois) and given water *ad libitum*.

A lipid emulsion containing 7,8,12-TMBA, 0·5% (w/v), was prepared by the method of Schurr (1969). The emulsion was injected into a caudal vein and the day of first injection designated Day 0. The leukaemic rats were chosen from a larger group of treated animals in which the total incidence of leukaemia was approximately 70%. Untreated littermates served as controls.

Heparinized blood for haematological studies was obtained by cardiac puncture. Leucocytes and erythrocytes were enumerated electronically (Coulter Counter Model Z, Coulter Electronics, Inc., Hialeah, Florida). Haemoglobin concentration was measured spectrophotometrically (Drabkin and Austin, 1935–36). Peripheral blood smears were fixed in 100% methanol and stained with Giemsa stain. For histological studies tissues were fixed in Bouin's solution and paraffin sections stained with haematoxylin and eosin.

The preparation of tissue homogenates for enzyme study has already been described (Bird and Huggins, 1971). After centrifugation, the supernatant was removed and kept at 4°C until enzyme assay was performed. Spectrophotometric determinations were made with a Beckman Model DU spectrophotometer using optical cells with a 1 cm light path. LDH (L-lactate: nicotinamide adenine dinucleotide (NAD) oxidoreductase, E.C.1.1.1.27) and MDH (L-malate: NAD oxidoreductase, E.C.1.1.1.37) activities were measured concurrently (Rees and Huggins, 1960). The initial velocity of the reaction was measured under conditions which yielded zero-order kinetics. One unit of LDH or MDH is defined as the enzyme activity which resulted in oxidation of 1 $\mu$mol of NADH in 1 min at 25°C. All enzyme units are expressed in terms of 1 g wet weight of tissue.

Tissue slices for metabolic studies were cut with a Stadie–Riggs microtome (Stadie and Riggs, 1944). Respiration was determined manometrically at 37°C in Warburg flasks containing 0·2 ml of 20% potassium hydroxide on a filter paper roll in the centre well. The slices were immersed in 2 ml of Krebs–Ringer phosphate solution (Umbreit, Burris and Stauffer, 1964) at pH 7·4 with 0·2% glucose, but without calcium. The gas phase was 100% $O_2$. Glycolysis was measured at 37° C in flasks containing 2 ml of Krebs–Ringer bicarbonate solution (Umbreit *et al.*, 1964), at pH 7·4 with 0·2% glucose, but without calcium. For aerobic glycolysis the gas phase was 95% $O_2$–5% $CO_2$ and for anaerobic glycolysis 95% $N_2$–5% $CO_2$. Glycolysis was estimated by measuring the amount of lactic acid (Barker and Summerson, 1941) evolved in the media. It was found that the rate of glycolysis diminished with time and the glycolytic values quoted were those obtained during the first 45 minutes after tissue slices were placed in the flasks. The reaction was terminated by removal of the tissue slices. At the end of each experiment the slices were rinsed in distilled water, blotted and dried in an oven at 105°C for 24 hours. The respiration value $QO_2$, represents the $\mu$l $O_2$ consumed during the first hour of measured respiration/mg dry weight of tissue. The glycolytic values, $Q_L^{O_2}$ and $Q_L^{N_2}$, represent the $\mu$g lactic acid evolved during the first 45 minutes of measured glycolysis/mg dry weight of tissue.

Separation of LDH isoenzymes was achieved with a Millipore Phoroslide electrophoresis system (Millipore Corporation, Bedford, Massachusetts). Separation was performed on cellulose acetate strips in Tris-barbitol buffer at pH 8·3. The enzyme was stained with phenazine methosulphate and nitroblue tetrazolium according to instructions given in Millipore Corporation Bulletin PS, 1969. Quantitation of the separated isoenzymes was performed with a Millipore Phoroscope densitometer.

Data are presented as mean $\pm$ standard deviation; statistical significance between means was determined by Student's $t$ test and a $P$ value $< 0{\cdot}05$ was considered significant.

### RESULTS

### *Haematological changes in leukaemia*

Leukaemia was evoked in 20 female rats with 4 intravenous pulse-doses of 7,8,12-TMBA, 30–35 mg/kg body weight, at 14-day intervals beginning at 50 days of age. Haematological studies were performed at age 148 $\pm$ 29 days when leukaemia was at an advanced stage. Ten untreated female rats aged 148 days served as controls.

In rats with leukaemia there was severe anaemia and the number of circulating erythrocytes was greatly reduced (Table I). However, there were no significant changes in mean corpuscular volume, mean corpuscular haemoglobin, or mean corpuscular haemoglobin concentration of erythrocytes. Leucocytosis, usually of moderate grade, occurred in most leukaemic rats although in a few instances leucocyte counts were within normal limits. Peripheral blood smears revealed the presence of variable numbers of large blast-like mononuclear cells, 12–20 $\mu$m in diameter. The nuclei of these cells were rich in chromatin and often contained several indistinct nucleoli; the cytoplasmic component was deeply basophilic. Morphologically, these cells closely resembled undifferentiated erythroblastic cells. More mature normoblasts and increased numbers of polychromatic erythrocytes occurred in all leukaemic rats although these varied greatly in number. No nucleated erythrocytic cells were seen in the peripheral blood of control rats. Differential leucocyte counts in leukaemic animals showed a proportionate reduction in the number of lymphocytes (Table I).

TABLE I.—*Haematological Changes in Female Rats with Leukaemia**

| | Leukaemia | Control |
|---|---|---|
| No. rats | 20 | 10 |
| Haematocrit % | $21{\cdot}3\pm8{\cdot}3$† | $43{\cdot}5\pm1{\cdot}4$ |
| Haemoglobin g/100 ml | $6{\cdot}8\pm2{\cdot}6$† | $13{\cdot}8\pm0{\cdot}6$ |
| Erythrocytes $\times 10^6$/mm$^3$ | $2{\cdot}88\pm1{\cdot}11$† | $5{\cdot}88\pm0{\cdot}39$ |
| Leucocytes $\times 10^3$/mm$^3$ | $26{\cdot}27\pm23{\cdot}10$† | $7{\cdot}09\pm1{\cdot}19$ |
| —Neutrophils | 23·2 | 24·1 |
| %—Lymphocytes | 58·6† | 72·1 |
| —Others‡ | 0·9 | 3·8 |
| —Blast cells | 17·2† | 0 |
| Normoblasts§ | $66{\cdot}9\pm105{\cdot}6$† | 0 |

* Mean values $\pm$ S.D.

† $P < 0{\cdot}001$.

‡ Eosinophils, monocytes and basophils.

§ No. normoblasts /100 leucocytes counted.

### *Morphological involvement of lymph nodes in leukaemia*

Leukaemia was elicited in 25 female rats with 4 intravenous pulse-doses of 7,8,12-TMBA, 30–35 mg/kg body weight, at 14-day intervals beginning at 50 days of age. Autopsy was performed at age 151 $\pm$ 31 days when all animals showed marked hepatomegaly. Fifteen untreated control female rats were autopsied at age 144 $\pm$ 37 days.

Four visceral and 2 superficial groups of lymph nodes were excised for histological study. They were designated and located as follows: (1) *coeliac*—in the retroperitoneal tissues surrounding the coeliac artery; (2) *mesenteric*—in the folds of the intestinal mesentery adjacent to the caecum; (3) *iliac*—in the retroperitoneal space adjacent to the aortic bifurcation; (4) *mediastinal*—lateral to the thymus; (5) *axillary*—in the loose fascia of the axilla; (6) *inguinal*—in the subcutaneous

TABLE II.—*Lymph Node Involvement in 25 Female Rats with Leukaemia*

| | No. rats with involved lymph nodes (%) | No. lymph nodes involved by leukaemia/ No. examined (%) | Weight of lymph node in leukaemic rat mg/100 g* (No. weighed) | Weight of lymph node in control rat mg/100 g* (No. weighed) |
|---|---|---|---|---|
| Coeliac | 25 | 101/151 | 3·9±2·9 | 3·1±0·8 |
| | (100) | (66·9) | (172) | (73) |
| Mediastinal | 15 | 35/108 | 3·3±1·8 | 3·4±1·3 |
| | (60) | (32·4) | (127) | (90) |
| Iliac | 6 | 8/86 | 2·5±1·0† | 4·2±1·0 |
| | (24) | (9·3) | (87) | (46) |
| Mesenteric | 1 | 1/130 | 4·9±1·7† | 8·5±2·3 |
| | (4) | (0·8) | (138) | (85) |
| Axillary | 0 | 0/51 | 4·2±1·7‡ | 7·2±3·3 |
| | | (0) | (54) | (62) |
| Inguinal | 0 | 0/42 | 2·9±1·1§ | 4·4±1·0 |
| | | (0) | (45) | (51) |

* Mean values ± standard deviation/100 g body weight.
† $P < 0{\cdot}001$.
‡ $P < 0{\cdot}01$.
§ $P < 0{\cdot}005$.

tissues of the groin. All lymph nodes were sectioned at multiple levels.

Leukaemic cell deposits were found in the coeliac group of lymph nodes of all leukaemic animals. Fifteen leukaemic rats (60%) also showed leukaemic infiltrates in mediastinal lymph nodes while iliac lymph nodes were involved in 6 leukaemic animals (24%). In one animal a deposit of leukaemic cells was found in one mesenteric lymph node but no animals showed involvement of axillary or inguinal lymph nodes (Table II). The proportion of individual lymph nodes involved by leukaemia showed a similar selective pattern of involvement. Thus, 67% of all coeliac, 32% of mediastinal, 9% of iliac and 0·8% of mesenteric lymph nodes were found to contain deposits of leukaemic cells (Table II). The earliest leukaemic cell deposits were found in the peripheral sinuses of lymph nodes, and later infiltration of deeper parts of the node occurred. In leukaemic rats the mean weight of coeliac and mediastinal lymph nodes was approximately equal to that of untreated controls but with other lymph node groups atrophy of lymphoid elements was observed and there was significant reduction in mean lymph node weight (Table II). Essentially similar changes were found in the lymph nodes of groups of male rats with leukaemia.

*LDH and MDH activity in lymphoid tissues*

Leukaemia was induced in 12 male rats with 4 intravenous doses of 7,8,12-TMBA, 30–35 mg/kg body weight, at 10-day intervals beginning at age 28 days. They were killed at age 120 ± 14 days. Ten untreated male control rats were killed at age 107 ± 7 days. Histological confirmation of leukaemia was obtained for each tissue examined biochemically.

Measurement of LDH and MDH activity was performed concurrently; it has been found useful to relate the activity of these enzymes as a quotient, $Q\,\frac{\text{LDH}}{\text{MDH}}$ (Reddi and Huggins, 1971).

In the lymphoid tissues of normal rats MDH activity always exceeded that of LDH, and the

$$Q\,\frac{\text{LDH}}{\text{MDH}} = < 1 \quad \text{(Table III).}$$

But in leukaemia, activity of LDH in spleen and coeliac lymph nodes was increased whereas MDH was reduced; thus

$$Q\,\frac{\text{LDH}}{\text{MDH}} = > 1 \quad \text{(Table III).}$$

By comparison, mesenteric lymph nodes of leukamic rats, which virtually never contained leukaemic infiltrates (Table II),

TABLE III.—*Dehydrogenase Activity in Lymphoid Tissues of Male Rats with Leukaemia**

| | Leukaemia | | | Control | | |
|---|---|---|---|---|---|---|
| | LDH | MDH | Q $\frac{LDH}{MDH}$ | LDH | MDH | Q $\frac{LDH}{MDH}$ |
| Coeliac lymph nodes | 104·7±24·8† | 95·5±20·2‡ | 1·11±0·24‡ | 80·4±5·9 | 142·2±8·9 | 0·57±0·04 |
| Mesenteric lymph nodes | 86·9±9·7 | 133·5±17·4 | 0·66±0·09 | 80·9±5·6 | 123·4±4·3 | 0·66±0·05 |
| Spleen | 107·9±24·4§ | 91·1±13·2‡ | 1·20±0·28‡ | 83·2±4·3 | 124·2±9·1 | 0·67±0·06 |
| Thymus | — | — | — | 96·8±9·6 | 135·9±10·5 | 0·71±0·06 |

* 12 leukaemic rats and 10 untreated male controls were studied.
Enzyme activity = units/g wet weight as defined. Mean values ± S.D. given.
† $P < 0·005$.
‡ $P < 0·001$.
§ $P < 0·01$.

showed no significant change in the normal levels of enzyme activity.

*Respiration, glycolysis and dehydrogenase activity of leukaemic spleen*

Leukaemia was induced in 10 female rats with 4 intravenous doses of 7,8,12-TMBA, 30–35 mg/kg body weight, at 14-day intervals beginning at age 50 days. Animals were killed at age 142 ± 30 days when leukaemia was at an advanced stage. Eight untreated female rats aged 122 ± 29 days served as controls.

The respiration values obtained for spleens of leukaemic rats were similar to those shown by untreated control rats (Table IV). However, in rats with leukaemia a marked increase in the rate of aerobic and anaerobic glycolysis was observed (Table IV).

*LDH isoenzymes in leukaemic spleen*

Leukaemia was induced in 8 female rats with 4 intravenous injections of 7,8,12-TMBA, 30–35 mg/kg body weight, at 14-day intervals beginning at age 50 days. Autopsy was performed at age 145 ± 27 days. Five untreated control female rats were autopsied at age 143 ± 29 days.

In the spleen of normal rats 4 molecular forms of LDH could be separated electro-

TABLE IV.—*Respiration, Glycolysis and Dehydrogenase Activity in Spleen of Female Rats with Leukaemia**

| | Leukaemia | Control |
|---|---|---|
| No. rats | 10 | 8 |
| $QO_2$† | 10·6±1·0 | 9·5±1·6 |
| $Q_L^{O_2}$‡ | 17·4±5·6¶ | 10·1±1·6 |
| $Q_L^{N_2}$‡ | 35·8±9·0¶ | 20·1±6·1 |
| LDH§ | 128·4±20·5‖ | 89·8±4·4 |
| MDH§ | 94·9±11·4‖ | 139·7±9·0 |
| Q $\frac{LDH}{MDH}$ | 1·37±0·26‖ | 0·65±0·03 |

* Mean values ± S.D. given.

† $QO_2$ = µl $O_2$ consumed/hr/mg dry weight.

‡ $Q_L^{O_2}$ and $Q_L^{N_2}$ = µg lactic acid evolved/45 min/mg dry weight.

§ Enzyme activity = units/g wet weight as defined.

‖ $P < 0·001$.

¶ $P < 0·005$.

TABLE V.—*Lactate Dehydrogenase Isoenzymes in Spleen of Female Rats with Leukaemia*

| | | | % Isoenzyme type | | | | |
|---|---|---|---|---|---|---|---|
| | No. rats | LDH* | I | II | III | IV | V |
| Leukaemia | 8 | 127·5±13·9† | 0 | 0·1 | 2·9 | 24·8 | 72·2† |
| Control | 5 | 88·7±6·2 | 0 | 2·1 | 11·8 | 30·8 | 55·3 |

* Enzyme activity = units/g wet weight as defined. Mean values ± S.D. given.
† $P < 0·001$.

phoretically: $LDH_2$, $LDH_3$, $LDH_4$, and $LDH_5$. In the spleen of rats with leukaemia there was a marked increase in the level of $LDH_5$, the most negatively migrating form, while other molecular forms were somewhat reduced (Table V).

### DISCUSSION

Concurrent measurement of LDH and MDH activity in tissues containing substantial infiltrates of leukaemic stem cells has revealed a striking change in the relative proportion of these critical enzymes. It has been shown previously that repeated hydrocarbon treatment by itself has little influence on the level of splenic dehydrogenase activity before leukaemia has evolved (Bird and Huggins, 1971). Therefore it seems reasonable to ascribe the changes in enzyme activity to intrinsic properties of the leukaemic stem cells. Moreover, Rees and Huggins (1960) have shown a similar change in the relative proportion of LDH and MDH in mammary cancers of rodents compared with the hyperplastic mammary glands of rats in pregnancy and lactation. LDH and MDH are essential enzymes for glycolytic and oxidative metabolic pathways of both normal and neoplastic tissues (Aisenberg, 1961). Since a high rate of glycolysis is a characteristic feature of virtually all rapidly growing cancers it was suggested (Bird and Huggins, 1971; Bird, 1972) that the alteration in the relative proportion of LDH and MDH might be attributed to a change in the metabolism of leukaemic tissues. We have shown in the experiments reported here, that the spleen of leukaemic rats exhibits a considerable increase in the normal rate of glycolysis both in the presence and absence of oxygen. Thus, the increased level of LDH activity correlates well with the elevation of glycolytic activity. Furthermore, a characteristic change was observed in the molecular forms of LDH in leukaemic spleen; the proportion of $LDH_5$, muscle (M)-type LDH, was greatly increased. A similar increase in M-type LDH has been observed in a large series of malignant human tumours (Goldman, Kaplan and Hall, 1964). It has been suggested that this molecular form of LDH is best adapted functionally for anaerobic metabolism (Cahn *et al.*, 1962); but a marked reduction of MDH activity was also observed in leukaemic tissues despite the fact that in leukaemic spleen no significant change occurred in the respiration rate. It is apparent, therefore, that concurrent measurements of LDH and MDH activity may not relate directly to the levels of tissue respiration and glycolysis estimated by conventional methods. Nevertheless, the striking alteration in the relative proportion of these enzymes in leukaemic tissues is a remarkably constant finding and while the precise significance of this change clearly awaits further elucidation, it is highly suggestive of some alteration in the metabolic characteristics of leukaemic tissues.

The selective nature of lymph node involvement in stem cell leukaemia is of interest. Coeliac lymph nodes greatly exceeded the other groups in their incidence of leukaemic involvement. Huggins and Froehlich (1966) also found, during a study of the distribution of injected titanium dioxide ($TiO_2$) in the rat, that coeliac lymph nodes appeared to possess a distinctive scavenging property which set them apart from other reticulo-endothelial tissues. However, the great accumulation of $TiO_2$ in coeliac nodes was attributed to their topography rather than unusual chemical characteristics since these nodes are the chief filters of hepatic lymph.

In the terminal stages of stem cell leukaemia induced with TMBA, the liver contains large numbers of leukaemic cells which filter first in hepatic lymph to coeliac lymph nodes. Thereafter, hepatic lymph drains through the cisterna chyli to the thoracic duct and thence to the jugular vein, receiving lymphatic tributaries in the abdomen and chest from the iliac and mediastinal lymph nodes. It is postulated that metastatic seeding of the leukaemic stem cells within these channels according to the predominant flow of lymph, accounts for the selective pattern of lymph node involvement in leukaemia. It was suggested previously (Huggins and Froehlich, 1966) that in lymphoblastic leukaemia of AK mice (Pollard, Kajima and Teah, 1965), where liver is frequently involved, that specific involvement of lymph nodes in the coeliac region might occur for a similar reason.

We thank Dr John Pataki, Ben May Laboratory for Cancer Research, University of Chicago, Illinois, for synthesis of 7,8,12-TMBA and Dr Paul E. Schurr, Upjohn Co., Kalamazoo, Michigan, for lipid emulsions. This work was supported by grants from the American Cancer Society, the Jane Coffin Childs Memorial Fund for Medical Research, and U.S. Public Health Service, National Institutes of Health (No. CA 11603).

### REFERENCES

AISENBERG, A. C. (1961) *The Glycolysis and Respiration of Tumours.* New York: Academic Press.

BARKER, S. B. & SUMMERSON, W. H. (1941) Colourimetric Determination of Lactic Acid in Biological Material. *J. biol. Chem.*, **138**, 535.

BIRD, C. (1972) Leukaemia Induced by 7,8,12-trimethylbenz(a)anthracene in Rat. II. Changes in Bone Marrow. *J. natn. Cancer Inst.*, **48**, 429.

BIRD, C. & HUGGINS, C. (1971) Leukaemia Evoked with 7,8,12-trimethylbenz(a)anthracene in Rat. I. Changes in Spleen and Thymus. *J. exp. Med.*, **134**, 1285.

CAHN, R. D., KAPLAN, N. O., LEVINE, L. & ZWILLING, E. (1962) Nature and Development of Lactic Dehydrogenases. *Science, Washington*, **136**, 962.

DRABKIN, D. L. & AUSTIN, J. H. (1935–36) Spectrophotometric Studies. II. Preparations from Washed Blood Cells: Nitric Oxide Haemoglobin and Sulphaemoglobin. *J. biol. Chem.*, **112**, 51.

GOLDMAN, R. D., KAPLAN, N. O. & HALL, T. C. (1964) Lactic Dehydrogenase in Human Neoplastic Tissues. *Cancer Res.*, **24**, 389.

HUGGINS, C. B. & FROEHLICH, J. P. (1966) High Concentration of Injected Titanium Dioxide in Abdominal Lymph Nodes. *J. exp. Med.*, **124**, 1099.

HUGGINS, C. B. & SUGIYAMA, T. (1966) Induction of Leukaemia in Rat by Pulse-doses of 7,12-dimethyl benz(a)anthracene. *Proc. natn. Acad. Sci. U.S.A.*, **55**, 74.

HUGGINS, C. B. & KUWAHARA, I. (1967) *Effect of Dexamethasone on Stem-cell Leukaemias of Rat. In Endogenous Factors Influencing Host–tumour Balance.* Ed. R. W. Wissler, T. L. Dao & S. Wood. University of Chicago Press. p. 9.

HUGGINS, C., GRAND, L. & OKA, H. (1970) Hundred Day Leukaemia: Preferential Induction in Rat by Pulse-doses of 7,8,12-trimethylbenz(a)anthracene. *J. exp. Med.*, **131**, 321.

POLLARD, M., KAJIMA, M. & TEAH, B. A. (1965) Spontaneous Leukaemia in Germ Free AK Mice. *Proc. Soc. exp. Biol. Med.*, **120**, 72.

REDDI, A. H. & HUGGINS, C. (1971) Lactic/malic Dehydrogenase Quotients during Transformation of Fibroblasts into Cartilage and Bone. *Proc. Soc. exp. Biol. Med.*, **137**, 127.

REES, E. D. & HUGGINS, C. (1960) Steroid Influences on Respiration, Glycolysis and Levels of Pyridine Nucleotide-linked Dehydrogenases of Experimental Mammary Cancers. *Cancer Res.*, **20**, 963.

REES, E. D., MAJUMDAR, S. K. & SHUCK, A. (1970) Changes in Chromosomes of Bone Marrow after Intravenous Injections of 7,12-dimethylbenz(a)-anthracene and Related Compounds. *Proc. natn. Acad. Sci. U.S.A.*, **66**, 1228.

SCHURR, P. E. (1969) Composition and Preparation of Experimental Intravenous Fat Emulsions. *Cancer Res.*, **29**, 258.

STADIE, W. C. & RIGGS, B. C. (1944) Microtome for Preparation of Tissue Slices for Metabolic Studies of Surviving Tissues *in vitro*. *J. biol. Chem.*, **154**, 687.

SUGIYAMA, T., KURITA, Y. & NISHIZUKA, Y. (1967) Chromosomal Abnormality in Rat Leukaemia Induced by 7,12-dimethylbenz(a)anthracene. *Science, Washington*, **158**, 1058.

SUGIYAMA, T., KURITA, Y. & NISHIZUKA, Y. (1969) Biologic Studies on 7,12-dimethylbenz(a)anthracene Induced Rat Leukaemia with Special Reference to the Specific Chromosomal Abnormalities. *Cancer Res.*, **29**, 1117.

SUGIYAMA, T. & BRILLANTES, F. P. (1970) Cytogenetic Studies of Leukaemia Induced by 6,8,12- and 7,8,12-trimethylbenz(a)anthracene. *J. exp. Med.*, **131**, 331.

UMBREIT, W. W., BURRIS, R. H. & STAUFFER, J. F. (1964) *Manometric Techniques*, 4th Ed. Minneapolis: Burgess. p. 131.

WARBURG, O., POSENER, K. & NEGELEIN, E. (1930) In *Metabolism of Tumours.* Ed. O. Warburg. London: Constable. p. 129.